# Supplementary material for: Spatial heterogeneity enhance robustness of large multi-species ecosystems
Source: PLoS Comput Biol. 2021 Oct 27;17(10):e1008899. doi: 10.1371/journal.pcbi.1008899 (PMC8575308; doi:10.1371/journal.pcbi.1008899)
Supplement: Attachment — Submitted filename: Reply to Spatial heterogeneity enhance robustness of large multi-species ecosystems.odt [file pcbi.1008899.s001.odt]

Dear Plos Computation Biology editorial team, 

I’m afraid that, while I find the results presented in the manuscript Spatial heterogeneity enhance robustness of large multi-species ecosystems of interest, I cannot recommend publication of this study in its current form. The major issues for me relate to the novelty of the results, the author’s representation of the study including the technical details, and the apparent absence of much of the relevant literature from the discussion. My arguments are as follows:

The authors show that synchrony in population fluctuations is maximised at high dispersal, in which case spatially extended systems can behave like a single oscillator. That the degree of synchrony may decay as a function of site separation is well known. For example a brief internet search turned up evidence from Sutcliffe, Thomas and Moss (1996) that synchrony in intraspecific population dynamics decays with distance in butterflies. Likewise, and also using butterflies as a study taxon, Ranta et al (1995) use population abundance time series to probe the Moran effect (1953), according to which synchrony in abundance fluctuations can arise in the absence of spatial connectivity due to environmental autocorrelation, making disentangling the spatial and environmental drivers of such synchrony a major challenge. In light of this literature I don’t think the correlation between diffusion rate and dynamical synchronicity is a novel observation.

The authors show that asynchrony in population fluctuations can be stabilising in multi-species metacommunities. Here, this is represented by a species level Portfolio effect (Doak et al. 1998). The Portfolio effect as typically envisaged relies on averaging across species such that community level properties remain temporally robust, often despite significant fluctuations at the population scale (Magurran and Henderson, 2010). In the present study, this averaging occurs across space and within metapopulations, therefore is not identical to the effect original proposed by Doak et al, however the absence of this rich literature in the manuscript is an important oversight.

The authors show that in the case of extremely limited dispersal, spatially resolved systems behave like point communities. This observation is described as a particular outcome of the present study, referred to in the abstract as and ‘additional’ outcome. To me this is not a novel result of the model proposed, but instead a useful reality check: I would fully expect that point community dynamics are returned in spatial models once dispersal is switched off.

I find the author’s representation of the assumptions of spatially unresolved models problematic. For example, that 0D community models make an “assumption of an average and equal amount of interaction for all individuals of a species” is rather unclear, both semantically, and conceptually. Furthermore, in describing this assumption as a key drawback to spatially implicit models, I expected this present model to include some spatial distribution in interaction coefficients or intrinsic growth rates which could vary the outcome of ecological interactions across space, however this is absent from the model. Instead the authors include interspecific differences in diffusion rates within a regular lattice or line arena. They observe self-organise spatial compositional heterogeneity once the standard deviation of interaction coefficients crosses some threshold, but in effect they make the same assumptions regarding species interactions as a 0D LV model. This emergent spatial structure is an interesting and potentially important outcome, but similar emergent structure has been reported recently (Litaud et al. 2019) and in the present manuscript this self-organisation process is almost completely overlooked. 

Experimental variation of the diffusion rate was the primary ‘treatment’ explored by the model. However it is not clear from the manuscript how this was achieved. The authors report that D_i were sampled independently for each species from a uniform distribution but give not further information on how the diffusion regime was varied. As this is key to their analysis, this information is essential. 

What the authors describe as a ‘Phase diagram’ is better labelled a ‘Parameter space’. This might seem pedantic but it’s critically important to concede where theoretical results are purely numerical; phase diagram implies analytic results which are not reported here. Furthermore, the sensitivity of the effect to variation within this space is seemingly rather limited. However, details on the range of parameter values are completely hidden, as are effects sizes, since the vertical and colour axes of the heat-map representations of this parameter space are not numerically labelled. As such the reader can’t distinguish between a difference of 1 or 1000% in the various emergent properties assessed.

Thus, while I thank the authors for their contribution, I cannot recommend publication of this study without a substantial update to the scientific background on which it leans, greater clarity on the technical aspects of the model system, and a reworking of the representation of the novelty of the results.

Doak, D. F., Bigger, D., Harding, E. K., Marvier, M. A., O'malley, R. E., & Thomson, D. (1998). The statistical inevitability of stability-diversity relationships in community ecology. The American Naturalist, 151(3), 264-276.

Magurran, A. E., & Henderson, P. A. (2010). Temporal turnover and the maintenance of diversity in ecological assemblages. Philosophical Transactions of the Royal Society B: Biological Sciences, 365(1558), 3611-3620.

Moran, P. A. P. (1953). The Statistical Analsis of the Canadian Lynx cycle. 1. Structure and Prediction. Australian Journal of Zoology, 1(2), 163-173.

Liautaud, K., van Nes, E. H., Barbier, M., Scheffer, M., & Loreau, M. (2019). Superorganisms or loose collections of species? A unifying theory of community patterns along environmental gradients. Ecology letters, 22(8), 1243-1252.

Ranta, E., Kaitala, V., Lindström, J., & Linden, H. (1995). Synchrony in population dynamics. Proceedings of the Royal Society of London. Series B: Biological Sciences, 262(1364), 113-118.

Sutcliffe, O. L., Thomas, C. D., & Moss, D. (1996). Spatial synchrony and asynchrony in butterfly population dynamics. Journal of Animal Ecology, 85-95.
