# Supplementary material for: Spatial heterogeneity enhance robustness of large multi-species ecosystems
Source: PLoS Comput Biol. 2021 Oct 27;17(10):e1008899. doi: 10.1371/journal.pcbi.1008899 (PMC8575308; doi:10.1371/journal.pcbi.1008899)
Supplement: Attachment — Submitted filename: ResponseToReviewers.docx [file pcbi.1008899.s002.docx]

Response to reviewers.

**To both reviewers**

We would like to thank both reviewers for their interest in our material but foremost for their very helpful suggestions of surrounding literature and research to put our own work in context in this complex and interesting field. These suggestions and associated comments have helped the manuscript immensely and we are very grateful! We hope that our new version now gives a more comprehensive background and context to present the novel findings of our own work, as well as more clarity on technical details. Below follow responses addressing each reviewer and their comments. References to lines (ex. L---) refer to the text in the “tracked changes” version of the manuscript.

**Reviewer 1**

Reviewer #1: The authors look at dynamics of interacting species (via the GLV equations), and add connected space to the system. The species interact in each location and are able to migrate between the different locations. They find that stability limits on GLV system when considered without space, are no longer restrictive due to the possibility of spatial heterogeneity, and the related appearance of unsynchronized oscillations of the abundances.

The manuscript looks well-written and draws clear and potentially interesting conclusions. But at the moment it is hard for me to judge the novelty of the work because it seems, as far as I can see, very closely related to the following publications:
* Roy, Felix, Matthieu Barbier, Giulio Biroli, and Guy Bunin. “Complex Interactions Can Create Persistent Fluctuations in High-Diversity Ecosystems.” PLOS Computational Biology 16, no. 5 (2020): e1007827.
* Pearce, Michael T., Atish Agarwala, and Daniel S. Fisher. “Stabilization of Extensive Fine-Scale Diversity by Ecologically Driven Spatiotemporal Chaos.” Proceedings of the National Academy of Sciences 117, no. 25 (2020): 14572–83.

In both these references, different locations in space with GLV (or replicator equations) are coupled via migration. Dynamics of the abundances appear and are argued to allow for stability bounds to be crossed. The theme of de-synchronization plays a role in these references as well, as does the non-trivial dependence on migration rates.
One difference between the present manuscript and these references, is that the locations in space are here placed on a grid versus all-to-all connections. But in the present manuscript the topology of the connections is claimed not to be very important to the qualitative results (when using 1d versus 2d or 3d grid).

It may very well be that the present manuscript contains novel results beyond these publications, but in order to make a judgement, I'd first like to see a comparison by the authors, between their work and the above references.

**Response:**

Great thanks to the reviewer for bringing these two publications to our attention! We agree that in the previous manuscript the novelty of our work compared to these publications is not at all clear.

The main novelty we find in our work is the increased robustness of spatially connected systems for a large range of interaction strengths (sigma), which does not rely on chaotic dynamics or heterogeneity in interaction between spatial patches. For example, a structural perturbation (represented by a perturbation in sigma) can in a connected system lead to species abundance oscillations unsynchronized or not, whereas in an unconnected system extinctions would occur. In contrast to the other studies (also reporting higher diversity) we find that this does not entail switching from a fixed point to completely different dynamics. Species abundances can remain almost unchanged for the entire system (asynchronized oscillations) or oscillate slightly (synchronized oscillations). The spatial extension allows the system to stay in the same “fixed point”, which in the unconnected system would be unstable. This is a previously unreported but relevant phenomenon. This same phenomenon can help the system cross previous stability boundaries, which (Roy et al., 2020) also reported but in their case for chaotic scenarios with interaction heterogeneity. We also find an increase in robustness due to the increased possibilities of system dynamics, including oscillations, combinations of different oscillation patterns, oscillations and fixed points and different fixed points across the grid. A previously unreported source of robustness. We believe that the increased robustness we find both in terms of asynchronous oscillations, oscillations and dynamical possibilities is highly relevant when interpreting the behavior of real ecosystems under pressure.

Both the studies mentioned have constant diffusion rates and all-to-all connections. In case of diffusion rates we chose to not vary over the grid but between species. This variation it turns out had little impact on dynamics, although it provided a very slight leaning towards asynchronous oscillations. This might be another source of robustness but needs more investigation with larger variation in diffusion rates. This finding is now mentioned in the manuscript (L225-232).

Difference in results between grid and all-to-all connections is not obvious. We did investigate the grid topology in different dimensions and found no qualitative differences (as noted by the reviewer). Simulations on different topologies done on a later date (for next article), has shown that qualitatively the same dynamics appear but at varying diffusion magnitudes due to the connectedness of the network. Without any conclusive results, a preliminary thought is that the same dynamics would appear on a grid and in all-to-all connections but at different diffusion magnitudes. Or, maybe, the grid set-up has a regime of low diffusion with a large spread of possible dynamics within the grid (feeding the mechanism of higher robustness) not available to the all-to-all topology due to the higher connectedness of the all-to-all. This we have not investigated in detail and is left for future studies.

In addition, there are differences in model details listed below.

Differences in model to (Roy et al., 2020) include:

- heterogeneous interaction matrices in different patches
- constant sigma (std(A))
- constant diffusion rates
- all-to-all connections

Differences in model to (Pearce et al., 2020) include:

- antisymmetric interaction matrices
- negative intrinsic growth rates r
- no intraspecific regulation/interaction
- constant diffusion rates
- all-to-all connections

We hope that the new version of the manuscript now makes our contribution to the field in comparison to the two previous studies clear! The change of focus from crossing stability limits to robustness is now included throughout the text but specifically in abstract, author summary, and discussion.

**Reviewer 2**

Dear Plos Computation Biology editorial team,

I’m afraid that, while I find the results presented in the manuscript *Spatial heterogeneity enhance robustness of large multi-species ecosystems* of interest, I cannot recommend publication of this study in its current form. The major issues for me relate to the novelty of the results, the author’s representation of the study including the technical details, and the apparent absence of much of the relevant literature from the discussion. My arguments are as follows:

- The authors show that synchrony in population fluctuations is maximised at high dispersal, in which case spatially extended systems can behave like a single oscillator. That the degree of synchrony may decay as a function of site separation is well known. For example a brief internet search turned up evidence from Sutcliffe, Thomas and Moss (1996) that synchrony in intraspecific population dynamics decays with distance in butterflies. Likewise, and also using butterflies as a study taxon, Ranta et al (1995) use population abundance time series to probe the Moran effect (1953), according to which synchrony in abundance fluctuations can arise in the absence of spatial connectivity due to environmental autocorrelation, making disentangling the spatial and environmental drivers of such synchrony a major challenge. In light of this literature I don’t think the correlation between diffusion rate and dynamical synchronicity is a novel observation.

**Response:** We expressed ourselves very confusing regarding synchronicity and diffusion in the previous version. The reviewer’s comment really made this clear! We did reference papers on synchronicity and diffusion ((Molofsky and Ferdy, 2005),(Earn et al., 2000), (Matter and Roland, 2010)) but on the other hand, in the discussion, we claimed that the drawbacks of high diffusion were not known (L287-291, L302-307). To remedy this lack of coherence and the impression that we are presenting synchronicity at high diffusion as a novelty we added a paragraph in the introduction (L44-51) regarding meta-community studies, dispersal and synchronicity, as well as removing the paragraph in the discussion (L287-291, L302-307). Our aim is not to present this effect as new but to put it in the stability/robustness context of our results for multispecies systems. We hope this is achieved in the new manuscript.

- The authors show that asynchrony in population fluctuations can be stabilising in multi-species metacommunities. Here, this is represented by a species level Portfolio effect (Doak et al. 1998). The Portfolio effect as typically envisaged relies on averaging across species such that community level properties remain temporally robust, often despite significant fluctuations at the population scale (Magurran and Henderson, 2010). In the present study, this averaging occurs across space and within metapopulations, therefore is not identical to the effect original proposed by Doak et al, however the absence of this rich literature in the manuscript is an important oversight.

**Response:** Great thanks to the reviewer for bringing this literature to our attention. As the reviewer mentions we are presenting a spatial version of the time-averaged Portfolio effect. Including this research really helps put our results in context! We added a paragraph in the discussion (L272-277) relating our work to their results.

- The authors show that in the case of extremely limited dispersal, spatially resolved systems behave like point communities. This observation is described as a particular outcome of the present study, referred to in the abstract as and ‘additional’ outcome. To me this is not a novel result of the model proposed, but instead a useful reality check: I would fully expect that point community dynamics are returned in spatial models once dispersal is switched off.

**Response:** Our intention with this point in the previous version (in the abstract for example) was not the insight itself but putting it in relation to the concept of fragmentation. We agree, it is an expected result and all such passages where we express this as a novelty in connection to fragmentation (abstract, author summery and conclusion) have been removed. Thanks for pointing this out!

- I find the author’s representation of the assumptions of spatially unresolved models problematic. For example, that 0D community models make an “assumption of an average and equal amount of interaction for all individuals of a species” is rather unclear, both semantically, and conceptually. Furthermore, in describing this assumption as a key drawback to spatially implicit models, I expected this present model to include some spatial distribution in interaction coefficients or intrinsic growth rates which could vary the outcome of ecological interactions across space, however this is absent from the model. Instead the authors include interspecific differences in diffusion rates within a regular lattice or line arena. They observe self-organise spatial compositional heterogeneity once the standard deviation of interaction coefficients crosses some threshold, but in effect they make the same assumptions regarding species interactions as a 0D LV model. This emergent spatial structure is an interesting and potentially important outcome, but similar emergent structure has been reported recently (Litaud et al. 2019) and in the present manuscript this self-organisation process is almost completely overlooked.

**Response:** We agree that our previous representation of spatially unresolved model assumptions was ambiguous. The model assumption regarding interactions and abundances is now rephrased as “spatial average of interactions among species and species abundances” (ex. L39-40). We hope that this representation is more explanatory!

The assumption we are removing in contrast to the spatially unresolved system is that of spatially averaged abundances. We leave room for spatial heterogeneity in abundances and thereby different dynamics in space. We see that the use of the expression “spatial heterogeneity” in the previous version was unlucky since, as the reviewer mentions, we have not added any differences in interactions, intrinsic growth rates etc. in space which is typically how other studies refer to spatial heterogeneity in their models. We are now more specific throughout the text that it is the possibility of spatial heterogeneity in abundances we are introducing (ex. in author summary and L100-101).

(Liautaud et al., 2019) have a similar set-up as ours but with a spatial gradient in terms of system parameters (sigma, mu and k) in their grid. As us, they find abrupt and smooth dynamical differences between local communities in the grid depending on diffusion rates, although they do not investigate stability aspects. This was an interesting and relevant read. We added this reference and discuss it in relation to our work (L308-309).

- Experimental variation of the diffusion rate was the primary ‘treatment’ explored by the model. However it is not clear from the manuscript how this was achieved. The authors report that D_i were sampled independently for each species from a uniform distribution but give not further information on how the diffusion regime was varied. As this is key to their analysis, this information is essential.

**Response:** A very helpful observation! We have now added the magnitudes of diffusion rates used in the text and a more careful explanation of how we vary the diffusion rates (L111-115).

- What the authors describe as a ‘Phase diagram’ is better labelled a ‘Parameter space’. This might seem pedantic but it’s critically important to concede where theoretical results are purely numerical; phase diagram implies analytic results which are not reported here. Furthermore, the sensitivity of the effect to variation within this space is seemingly rather limited. However, details on the range of parameter values are completely hidden, as are effects sizes, since the vertical and colour axes of the heat-map representations of this parameter space are not numerically labelled. As such the reader can’t distinguish between a difference of 1 or 1000% in the various emergent properties assessed.

**Response:** We have changed “Phase diagram” to “Parameter space”. To make our results more clear and help the reader we have updated Fig. 4 by additional statistics from 5 more runs, numerical values to the diffusion scale, increasing the colour-range in the diagrams and associated heat-maps, as well as more descriptive labels for the heat-maps. We agree that the previous version was vague. However, we decided not to introduce more numerical values on the heat-maps, since we wish to illustrate patterns and believe that numerical values (for the amplitude for example which we can understand is perceived as vague) only introduces unnecessary details confusing the purpose.

Thus, while I thank the authors for their contribution, I cannot recommend publication of this study without a substantial update to the scientific background on which it leans, greater clarity on the technical aspects of the model system, and a reworking of the representation of the novelty of the results.

**Final comment:** We are deeply grateful for the reviewer’s comments, we believe they have substantially improved the manuscript!

Doak, D. F., Bigger, D., Harding, E. K., Marvier, M. A., O'malley, R. E., & Thomson, D. (1998). The statistical inevitability of stability-diversity relationships in community ecology. The American Naturalist, 151(3), 264-276.

Magurran, A. E., & Henderson, P. A. (2010). Temporal turnover and the maintenance of diversity in ecological assemblages. Philosophical Transactions of the Royal Society B: Biological Sciences, 365(1558), 3611-3620.

Moran, P. A. P. (1953). The Statistical Analsis of the Canadian Lynx cycle. 1. Structure and Prediction. Australian Journal of Zoology, 1(2), 163-173.

Liautaud, K., van Nes, E. H., Barbier, M., Scheffer, M., & Loreau, M. (2019). Superorganisms or loose collections of species? A unifying theory of community patterns along environmental gradients. *Ecology letters*, *22*(8), 1243-1252.

Ranta, E., Kaitala, V., Lindström, J., & Linden, H. (1995). Synchrony in population dynamics. Proceedings of the Royal Society of London. Series B: Biological Sciences, 262(1364), 113-118.

Sutcliffe, O. L., Thomas, C. D., & Moss, D. (1996). Spatial synchrony and asynchrony in butterfly population dynamics. *Journal of Animal Ecology*, 85-95.

References

EARN, D. J., LEVIN, S. A. & ROHANI, P. 2000. Coherence and conservation. *Science,* 290**,** 1360-1364.

LIAUTAUD, K., VAN NES, E. H., BARBIER, M., SCHEFFER, M. & LOREAU, M. 2019. Superorganisms or loose collections of species? A unifying theory of community patterns along environmental gradients. *Ecology letters,* 22**,** 1243-1252.

MATTER, S. F. & ROLAND, J. 2010. Local extinction synchronizes population dynamics in spatial networks. *Proceedings of the Royal Society B: Biological Sciences,* 277**,** 729-737.

MOLOFSKY, J. & FERDY, J.-B. 2005. Extinction dynamics in experimental metapopulations. *Proceedings of the National Academy of Sciences,* 102**,** 3726-3731.

PEARCE, M. T., AGARWALA, A. & FISHER, D. S. 2020. Stabilization of extensive fine-scale diversity by ecologically driven spatiotemporal chaos. *Proceedings of the National Academy of Sciences,* 117**,** 14572-14583.

ROY, F., BARBIER, M., BIROLI, G. & BUNIN, G. 2020. Complex interactions can create persistent fluctuations in high-diversity ecosystems. *PLoS computational biology,* 16**,** e1007827.
