# Supplementary material for: Spatial heterogeneity enhance robustness of large multi-species ecosystems
Source: PLoS Comput Biol. 2021 Oct 27;17(10):e1008899. doi: 10.1371/journal.pcbi.1008899 (PMC8575308; doi:10.1371/journal.pcbi.1008899)
Supplement: Attachment — Submitted filename: Reply to Spatial heterogeneity enhance robustness of large multi-species ecosystems resubmission.odt [file pcbi.1008899.s003.odt]

Dear Plos Computation Biology editorial team, 

The authors have shown that the inclusion of spatial extent can permit the stable (persistent, robust) coexistence in ecological models with parameters for which feasible coexistence without spatial structure is impossible. Thus, the authors argue, that spatial structure buffers ecological communities from the effects of intrinsic regulatory processes (diversity limits) even in the absence of abiotic heterogeneity. This observation is interesting and timely and therefore I believe could be suitable for publication in PLoS computational biology. I thank the authors for addressing the technical and contextual issues I raised in the previous review, however there remain a number of issues that if addressed could improve the manuscript. 

I write this review as I am preparing to go on annual leave so I apologise if it seems rather rushed. Note, all line numbers refer to the version with changes tracked.

That non-uniform spatial structure, spatial beta diversity, emerges with no abiotic heterogeneity, is a key outcome of the study but one that receives relatively little attention in the manuscript. I understand that the authors are more concerned with the temporal aspects of the model, however I suggest the authors include some description, possibly with a figure, of the emergent spatial structure of their model. Given that much of the modelling was done in 1D space, this would be easy to represent graphically (see Fig 1b Litaud et al. 2019) and would help tie together the spatial and temporal aspects of the model.
I strongly suggest a reworking of the terminology used to describe species abundances. Abundance =/= population. Likewise, it is very confusing to use the same term for the 0D and the spatial models. There are multiple instances throughout the text and figures in which ‘species abundance’ is used to describe either the global abundance or spatial average in the spatial model, as well as the point abundance in the non-spatial case. The quantity of interest should always be explicit both linguistically and algebraically (this is true for Fig 3 but not for Fig 2, where the two model paradigms are contrasted). I suggest a switch to the ‘population-metapopulation’ terminology or some similar formulation, and that this terminology is used every time abundance is discussed.
I suggest some brief discussion of the fact that the inclusion of space is sufficient for simple models to predict persistent, feasible local population fluctuations, up to temporal compositional turnover (https://www.nature.com/articles/s41467-021-23769-7), an observation which could be of importance in biological monitoring.

Detailed comments:

L6: ‘ex.’ → ‘e.g.’ 
L11 and elsewhere: ‘acts (de)stabilising’ → ‘acts to (de)stabilise’ 
L16: ‘1970:ies’ → ‘1970s’ or ‘nineteen seventies’
L 16: ‘uni-vocal’ → ‘unanimous’
L17: Suggest single clarification that ‘diversity’ is used in the manuscript as synonymous to ‘Species richness’ since there are multiple types of ‘diversity’
L19: Suggest adding reference to mean interaction strength since both mean and variance determine diversity limits. It’s true that this is not relevant when sampling A_ij from a symmetric distribution, however this is a special case.
L22: Suggest ‘non-extinct’ → ‘extant’ or formal definition (abundance > 0)
L48-49: ‘Synchronise abundances’ is a little unclear, it may help to refer to ‘synchronise fluctuations in abundance’ so the reader knows that the synchrony is along a temporal axis.
L54-55: Ref 25 shows that the inclusion of greater area/environmental heterogeneity increases regional diversity limits and can thus be understood and increasing robustness relative to point (or indeed small meta) communities. This sentence seems mis-representative.
L62 ‘community disposition’ is unclear. Suggest ‘compositional difference between neighbouring communities’ or similar.
L78: interaction → interactions
L88-90: This sentence is unclear. Suggest phrases such as ‘Under increases in sigma’ and ‘threshold’ or similar. Sigma missing from L90
L103: Suggest replace ‘migrating’ with ‘occupying/colonising’ since ‘migration’ suggests a seasonal dispersal process.
L111-115: Suggest more formal representation of the sampling procedure for total clarity e.g. “$D_i~\mathcal{U}[0,2x10^a]$ with a, a parameter which we varied from -5 to 0 in steps of…” or similar.
L112: remove ‘a’
L113: ‘fom’ typo.
L121: Suggest ‘nearest neighbour sites/grid-points’ for clarity
L146-150: I believe there are some errors in terminology in this passage. When perturbed by increasing sigma, the spatial system transitions to a limit cycle; when these limit cycles are unsynchronised the global average abundance is effectively identical to the 0D case at intermediate sigma. The qualitative change in system dynamics that occurs may be effectively undetectable at the system scale, but this does not mean no change has occurred. Structural instability is a categorical property of a dynamical system though it is often described as a continuous property to which adjectives like ‘more’ or ‘less’ can be applied. So I understand that describing the spatial model as “more structurally stable”, while not entirely correct, is a common short-hand. However, if the stabilising effect of space is mediate through a transition from fixed point, to unsynchronised fluctuations, then this may be pushing the limits of the ‘structurally stable’ terminology. In this sense then I suggest further unpacking the phrases “structural stable (robustness)” and ‘ “stay” in the fixed point’ in particular regarding the spatial scales at which these descriptions apply, or don’t.
L159: This notation is a bit ambiguous since random draws from a uniform distribution can span multiple orders of magnitude. Suggest $D_i~\mathcal{U}[0,2x10^-2] or similar.
L160: If this means metapopulation abundances are stable (time invariant) the ‘metapopulation’ terminology could be useful in place of ‘species abundances of the whole ecosystem’ which suggests ‘total assemblage abundance’.
L165: Again if this refers to the global/spatial average abundance this should be made clear.
L166: Variability can mean a lot of things, perhaps the meaning in the present context can be clarified e.g. lower coefficient of variance in abundance over time or similar. Please also make clear if this variability is in average (or total) abundance in the spatial case.
L178: Consider describing these periodic local extinctions in terms of the ‘patch dynamics’ literature
L186: see 159 above.
L211; D_i=0, perhaps add (for all i) since  D_i is a scalar.
L199-201: These are not easily matched to the panels in Fig 3. Please consider adding panel numbers/letters to the figure and referencing these in the text. Also: 
The average number of grid points with oscillatory patterns (presumably after some numerical thresholding – if so please clarify). In Fig 3, panel 1 represents the percentage of grid cells not the average. This needs to be clearer.
Average phase shift. There are various metric of synchrony out there. Please clarify how synchrony is computed ideally early in the analysis methods since this is a key aspect of the analysis. In figure 3 the colour scale represents the percent of synchronised grids (grid points?). Does this mean the percent of grid points for which synchrony is \approx 1? Please clarify.
L225-232: While I understand that a detailed description of the impact of D_i/max(D_i) is beyond the current scope, if there are intuitive relationships between D_i/max(D_i) and the spatial distribution in abundance, it would be informative to discuss this. 
L234: It seems a little tenuous to describe May’s model as a representation of an ecosystem, and I am not sure that this was the first mathematical model in theoretical ecology. It would be better to pose this polemic by reference to the contradictory theoretical predictions arrived at by May and MacArthur on the issue of the complexity-stability debate.
L239: ‘Collapse’ is a misnomer here: perturbed ecological communities that exist near stability limits may lose a small proportion of their diversity.
L250: Please clarify ‘patterns’
L256-261: This passage is unclear to me. It seems to suggest that in completely homogeneous environments conspecific aggregation arises due to ‘attraction’, however intraspecific interactions in GLV models are strictly negative. 
L269: Again some revision of the terminology would make this much clearer, here the phrase ‘species populations as a whole’ is used to describe global, regional, or metapopulation abundance.
L321-322: see 19 above, they may differ in order.

Fig 2. In line with points made above, it would be helpful to use a different notation/terminology for the upper figure. You chose to report the average rather than total species abundance for the spatial model presumably to retain the same y axis scaling. I would suggest either a switch to total abundance with axis labels ‘$\sum_{x} x_{ix}$ (Metapopulation abundance)’ or retain the normalisation and include this in the axis title e.g. ‘$1/\sum_{x} x_{ix}$ (Spatial mean abundance)’ which has been used in Fig 3.
Fig 3. The vertical figure titles are confusing here since they appear to be axis titles, however this is not the case. The vertical axis title for all panels would be “Diffusion (order of mean)” or similar. I suggest moving these titles to a position above each panel rather than to the left to make them easier to interpret. It’s also odd that D_i is represented as a decreasing scale, and the the colour scale is does not have a consistent direction. In panel one, white represents the largest observed % grid-points undergoing oscillations. In panels 2 red represents high ‘% grids synchronised’. I suggest greater consistency with red representing high values in all cases.
