# Supplementary material for: Spatial heterogeneity enhance robustness of large multi-species ecosystems
Source: PLoS Comput Biol. 2021 Oct 27;17(10):e1008899. doi: 10.1371/journal.pcbi.1008899 (PMC8575308; doi:10.1371/journal.pcbi.1008899)
Supplement: Attachment — Submitted filename: ReplyToReviewers.docx [file pcbi.1008899.s004.docx]

Response to reviewers

We thank reviewer 2 for the new comments and suggestions to improve our manuscript! Below follow our replies to the reviewer’s comments, all line references refer to the new version with tracked changes.

- That non-uniform spatial structure, spatial beta diversity, emerges with no abiotic heterogeneity, is a key outcome of the study but one that receives relatively little attention in the manuscript. I understand that the authors are more concerned with the temporal aspects of the model, however I suggest the authors include some description, possibly with a figure, of the emergent spatial structure of their model. Given that much of the modelling was done in 1D space, this would be easy to represent graphically (see Fig 1b Litaud et al. 2019) and would help tie together the spatial and temporal aspects of the model.

**Reply:** To highlight the emergent spatial structure an extra figure is now included in the beginning of the results section showing the spatial structure at varying diffusion rates. In this section we describe spatial structure at increasing diffusion rates (combinations of fixed points and oscillations at low diffusion, synchronisation at high diffusion etc). The figure helps to get a better picture of this process!

- I strongly suggest a reworking of the terminology used to describe species abundances. Abundance =/= population. Likewise, it is very confusing to use the same term for the 0D and the spatial models. There are multiple instances throughout the text and figures in which ‘species abundance’ is used to describe either the global abundance or spatial average in the spatial model, as well as the point abundance in the non-spatial case. The quantity of interest should always be explicit both linguistically and algebraically (this is true for Fig 3 but not for Fig 2, where the two model paradigms are contrasted). I suggest a switch to the ‘population-metapopulation’ terminology or some similar formulation, and that this terminology is used every time abundance is discussed.

**Reply:** Thanks for these observations and suggestions. We have now consistently throughout the text and in figures used the terminology local and global species abundances for the spatial GLV, and non-spatial species abundances for the non-spatial GLV (regular GLV). If we are using the global average abundances we explicitly say so, when for example comparing to the non-spatial abundances in Fig. 2. We hope now that these changes make the text less confusing and our results clearer.

- I suggest some brief discussion of the fact that the inclusion of space is sufficient for simple models to predict persistent, feasible local population fluctuations, up to temporal compositional turnover (https://www.nature.com/articles/s41467-021-23769-7), an observation which could be of importance in biological monitoring.

**Reply:** Thanks for suggesting the angle of biological monitoring! We agree that understanding the baseline of intrinsic system dynamics is important for ecosystem monitoring and assessment. This is now discussed in an extra paragraph in the discussion (L315-325).

We thank the reviewer for the attention to detail in the comments below! Some of the reviewer points have no comments, they were simply implemented. Line numbers refer to the tracked changes document.

1. L6: ‘ex.’ → ‘e.g.’

2. L11 and elsewhere: ‘acts (de)stabilising’ → ‘acts to (de)stabilise’

3. L16: ‘1970:ies’ → ‘1970s’ or ‘nineteen seventies’

4. L 16: ‘uni-vocal’ → ‘unanimous’

5. L17: Suggest single clarification that ‘diversity’ is used in the manuscript as synonymous to ‘Species richness’ since there are multiple types of ‘diversity’ **Reply:** added clarification L13-14

6. L19: Suggest adding reference to mean interaction strength since both mean and variance determine diversity limits. It’s true that this is not relevant when sampling A_ij from a symmetric distribution, however this is a special case. **Reply:** We added reference to mean (L20). The mean does affect the collapse value, although not in as “simple” a way as the variance and diversity.

7. L22: Suggest ‘non-extinct’ → ‘extant’ or formal definition (abundance > 0)

8. L48-49: ‘Synchronise abundances’ is a little unclear, it may help to refer to ‘synchronise fluctuations in abundance’ so the reader knows that the synchrony is along a temporal axis.

9. L54-55: Ref 25 shows that the inclusion of greater area/environmental heterogeneity increases regional diversity limits and can thus be understood and increasing robustness relative to point (or indeed small meta) communities. This sentence seems mis-representative.

**Reply:** These sentences should primarily be referring to ref 24, this is now fixed in L47-49 to not misrepresent. Although on a more private note we wonder if it could be the case for ref 25 also. In previous work (with spatially unresolved systems) we have noticed that the structural instability is more “global” in the diversity equilibrium limit (at may’s limit), in the sense that many eigenvalues of the jacobian are close to zero. Could not an increase in the variance of the interaction strengths then lead to collapse/substantial loss of species? Although how such a perturbation translates to the meta-community picture is not clear.

10. L62 ‘community disposition’ is unclear. Suggest ‘compositional difference between neighbouring communities’ or similar.

11. L78: interaction → interactions

12. L88-90: This sentence is unclear. Suggest phrases such as ‘Under increases in sigma’ and ‘threshold’ or similar. Sigma missing from L90 **Reply:** This sentence is now rephrased L79-81.

13. L103: Suggest replace ‘migrating’ with ‘occupying/colonising’ since ‘migration’ suggests a seasonal dispersal process.

14. L111-115: Suggest more formal representation of the sampling procedure for total clarity e.g. “$D_i~\mathcal{U}[0,2x10^a]$ with a, a parameter which we varied from -5 to 0 in steps of…” or similar. **Reply:** Changed to more formal representation (L105-109) with the mean and standard deviation of the uniform distribution. Mean and s.d. is maybe not the usual way to represent the Uniform distribution but fits better with how we varied the D_i.

15. L112: remove ‘a’

16. L113: ‘fom’ typo.

17. L121: Suggest ‘nearest neighbour sites/grid-points’ for clarity

18. L146-150: I believe there are some errors in terminology in this passage. When perturbed by increasing sigma, the spatial system transitions to a limit cycle; when these limit cycles are unsynchronised the global average abundance is effectively identical to the 0D case at intermediate sigma. The qualitative change in system dynamics that occurs may be effectively undetectable at the system scale, but this does not mean no change has occurred. Structural instability is a categorical property of a dynamical system though it is often described as a continuous property to which adjectives like ‘more’ or ‘less’ can be applied. So I understand that describing the spatial model as “more structurally stable”, while not entirely correct, is a common short-hand. However, if the stabilising effect of space is mediate through a transition from fixed point, to unsynchronised fluctuations, then this may be pushing the limits of the ‘structurally stable’ terminology. In this sense then I suggest further unpacking the phrases “structural stable (robustness)” and ‘ “stay” in the fixed point’ in particular regarding the spatial scales at which these descriptions apply, or don’t.

**Reply:** A valid point. Our focus was on the global species abundances for the remark on structural stability, but with closer reflection a transition from local fixed points to local species oscillation is a qualitative change worth noting and maybe too great a change for the system to be called structurally stable. We now refrain from calling the system structurally stable and instead use robust in terms of extinction and global abundances. (L152-158)

19. L159: This notation is a bit ambiguous since random draws from a uniform distribution can span multiple orders of magnitude. Suggest $D_i~\mathcal{U}[0,2x10^-2] or similar.

**Reply:** We added a more formal description with the mean and standard deviation to clarify in this (L168) and all similar instances. With the standard deviation it is clear that the diffusion constants are kept within the same order of magnitude for each run. Thanks for noting this omission on orders of magnitude in our presentation of D_i.

20. L160: If this means metapopulation abundances are stable (time invariant) the ‘metapopulation’ terminology could be useful in place of ‘species abundances of the whole ecosystem’ which suggests ‘total assemblage abundance’.

21. L165: Again if this refers to the global/spatial average abundance this should be made clear.

**Reply 20 and 21:** phrases like ‘species abundances of the whole ecosystem’ are changed to ‘global species abundance’ throughout the text!

22. L166: Variability can mean a lot of things, perhaps the meaning in the present context can be clarified e.g. lower coefficient of variance in abundance over time or similar. Please also make clear if this variability is in average (or total) abundance in the spatial case.

**Reply:** Variability both regarding what the term refers to and if we apply it to the global or local scale is now clarified L175-180.

23. L178: Consider describing these periodic local extinctions in terms of the ‘patch dynamics’ literature

**Reply:** We have now clarified the sentences regarding variability and local extinctions and phrased it in line with patch-dynamics literature (L188-195).

24. L186: see 159 above.

25. L211; D_i=0, perhaps add (for all i) since D_i is a scalar.

26. L199-201: These are not easily matched to the panels in Fig 3. Please consider adding panel numbers/letters to the figure and referencing these in the text. Also:

- The average number of grid points with oscillatory patterns (presumably after some numerical thresholding – if so please clarify). In Fig 3, panel 1 represents the *percentage* of grid cells not the average. This needs to be clearer.

- Average phase shift. There are various metric of synchrony out there. Please clarify how synchrony is computed ideally early in the analysis methods since this is a key aspect of the analysis. In figure 3 the colour scale represents the percent of synchronised grids (grid points?). Does this mean the percent of grid points for which synchrony is \approx 1? Please clarify.

**Reply:** We added panel letters and coordinated the measures with titles in fig 4.

- It is the number of grid-points for all runs which have oscillations we are measuring. This is now changed in the text L215
- We have now added a paragraph in the theory and method section where we describe how synchronisation is measured (L121-128). We have also switched back to synchronised/unsynchronised nomenclature in figure 4 (the percentages seemed more confusing than helpful). We hope that the better explanation of how synchrony is measured now helps clarify the figure also.

27. L225-232: While I understand that a detailed description of the impact of D_i/max(**D**_i) is beyond the current scope, if there are intuitive relationships between D_i/max(**D**_i) and the spatial distribution in abundance, it would be informative to discuss this.

**Reply:** No intuitive relationships of this sort were found. No change in the oscillation patterns or fixed-point abundances in the patches were noticed in the dynamics. Rather, runs with different D_i draws with same initial abundances lead to qualitatively the same dynamics but with phase-shifts and high/low amplitudes in varying patches. We detected no pattern in how phases shifted etc. The specific D_i of species did not seem to have impact on these changes, rather the fact that there was a spread in the diffusion rates generated differences between runs overall.

28. L234: It seems a little tenuous to describe May’s model as a representation of an *ecosystem*, and I am not sure that this was the first mathematical model in theoretical ecology. It would be better to pose this polemic by reference to the contradictory theoretical predictions arrived at by May and MacArthur on the issue of the complexity-stability debate.

**Reply:** Although the title of May’s paper refers to large complex systems, he does talk of them applied in the ecological context, thus is not May’s model a representation of an ecosystem? Evan though he admits that random interactions are unrealistic. Nevertheless, maybe a little tenuous to phrase it as we did. We rewrote these sentences, instead referring to May’s mathematical argument for the instability of large complex systems and contrasting it to McArthur’s view that systems with higher complexity are more stable.

29. L239: ‘Collapse’ is a misnomer here: perturbed ecological communities that exist near stability limits may lose a small proportion of their diversity.

**Reply:** “radically change or collapse” -> “radically change, lose species or collapse”. Our previous work, when taking feasibility into account, has shown that the systems that do traverse May’s limit are no longer completely random and rather collapse/lose a substantial amount of species than just a few. But since we are here rather referring to the original formulation of the limit, we agree with the reviewer that a softer formulation is in order.

30. L250: Please clarify ‘patterns’. Patterns -> ‘carrying capacities and intrinsic growth rates’

31. L256-261: This passage is unclear to me. It seems to suggest that in completely homogeneous environments conspecific aggregation arises due to ‘attraction’, however intraspecific interactions in GLV models are strictly negative.

**Reply:** This passage was meant to give motivation for a homogeneous landscape to be “patchy” with different local populations. The individuals within the patches interact more with each other than with individuals of the same species in other patches. Thus, what we meant to mediate is that the patchiness of the model could be due to conspecific aggregation, while in the patches species interact according to GLV.

32. L269: Again some revision of the terminology would make this much clearer, here the phrase ‘species populations as a whole’ is used to describe global, regional, or metapopulation abundance. **Reply:** changed to global species abundances.

33. L321-322: see 19 above, they may differ in order. Ambiguity fixed by explicit reference to standard deviation of diffusion rates.

34. Fig 2. In line with points made above, it would be helpful to use a different notation/terminology for the upper figure. You chose to report the average rather than total species abundance for the spatial model presumably to retain the same y axis scaling. I would suggest either a switch to total abundance with axis labels ‘$\sum_{x} x_{ix}$ (Metapopulation abundance)’ or retain the normalisation and include this in the axis title e.g. ‘$1/\sum_{x} x_{ix}$ (Spatial mean abundance)’ which has been used in Fig 3.

**Reply:** We keep the scaling and have now changed the axis labels for the spatial case in line with the labels in figure 3 (mean spatial abundances). We hope this will clarify what the figures show!

35. Fig 4. Please consider adding panel numbers/letters to the figure and referencing these in the text. The vertical figure titles are confusing here since they appear to be axis titles, however this is not the case. The vertical axis title for all panels would be “Diffusion (order of mean)” or similar. I suggest moving these titles to a position above each panel rather than to the left to make them easier to interpret. It’s also odd that D_i is represented as a decreasing scale, and the the colour scale is does not have a consistent direction. In panel one, white represents the largest observed % grid-points undergoing oscillations. In panels 2 red represents high ‘% grids synchronised’. I suggest greater consistency with red representing high values in all cases.

**Reply:** All very good comments to clarify figure 4! We have now added letters in the text and specifically refer to panels by letters in the figure text. The vertical titles are now above and we have added an overall vertical title (Diffusion (order of mean and s.d.)). The diffusion scales are kept in the cryptic “D~1”-style in the figure but with clarification in the figure text. The diffusion now has an increasing scale in the panels and the colour scale is now consistent. More statistics is also added.

Again, we sincerely thank the reviewer for all suggestions and comments which have helped to improve the manuscript further.
